# The Relevance of Aquaporins for the Physiology, Pathology, and Aging of the Female Reproductive System in Mammals

**DOI:** 10.3390/cells9122570

**Published:** 2020-12-01

**Authors:** Paweł Kordowitzki, Wiesława Kranc, Rut Bryl, Bartosz Kempisty, Agnieszka Skowronska, Mariusz T. Skowronski

**Affiliations:** 1 Department of Basic and Preclinical Sciences, Institute for Veterinary Medicine, Nicolaus Copernicus University, 87-100 Torun, Poland; p.kordowitzki@umk.pl; 2 Institute of Animal Reproduction and Food Research of Polish Academy of Sciences, 10-243 Olsztyn, Poland; 3 Department of Anatomy, Poznan University of Medical Sciences, 60-781 Poznan, Poland; wiesiakranc@o2.pl (W.K.); rutbryl@gmail.com (R.B.); bkempisty@ump.edu.pl (B.K.); 4 Department of Histology and Embryology, Poznan University of Medical Sciences, 60-781 Poznan, Poland; 5 Department of Veterinary Surgery, Institute for Veterinary Medicine, Nicolaus Copernicus University, 87-100 Torun, Poland; 6 Department of Human Physiology and Pathophysiology, School of Medicine, Collegium Medicum, University of Warmia and Mazury, Warszawska Street 30, 10-082 Olsztyn, Poland; agnieszka.skowronska@uwm.edu.pl

**Keywords:** female reproductive system, aquaporin, physiology, connexin, gap-junctions, mammals, aging, ovary, uterus, placenta

## Abstract

Aquaporins constitute a group of water channel proteins located in numerous cell types. These are pore-forming transmembrane proteins, which mediate the specific passage of water molecules through membranes. It is well-known that water homeostasis plays a crucial role in different reproductive processes, e.g., oocyte transport, hormonal secretion, completion of successful fertilization, blastocyst formation, pregnancy, and birth. Further, aquaporins are involved in the process of spermatogenesis, and they have been reported to be involved during the storage of spermatozoa. It is noteworthy that aquaporins are relevant for the physiological function of specific parts in the female reproductive system, which will be presented in detail in the first section of this review. Moreover, they are relevant in different pathologies in the female reproductive system. The contribution of aquaporins in selected reproductive disorders and aging will be summarized in the second section of this review, followed by a section dedicated to aquaporin-related proteins. Since the relevance of aquaporins for the male reproductive system has been reviewed several times in the recent past, this review aims to provide an update on the distribution and impact of aquaporins only in the female reproductive system. Therefore, this paper seeks to determine the physiological and patho-physiological relevance of aquaporins on female reproduction, and female reproductive aging.

## 1. Introduction

**Figure 1:**
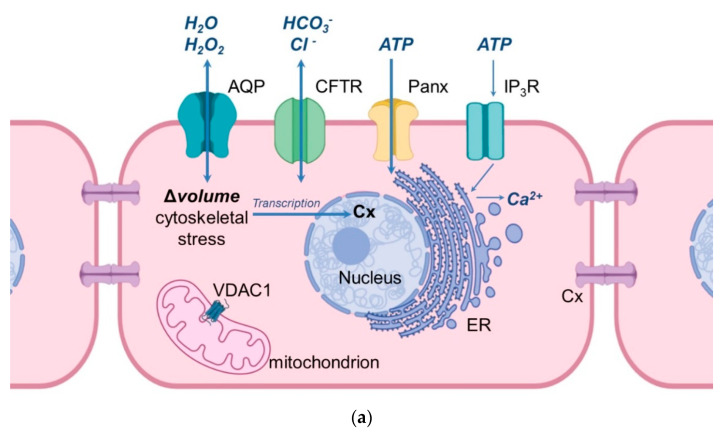

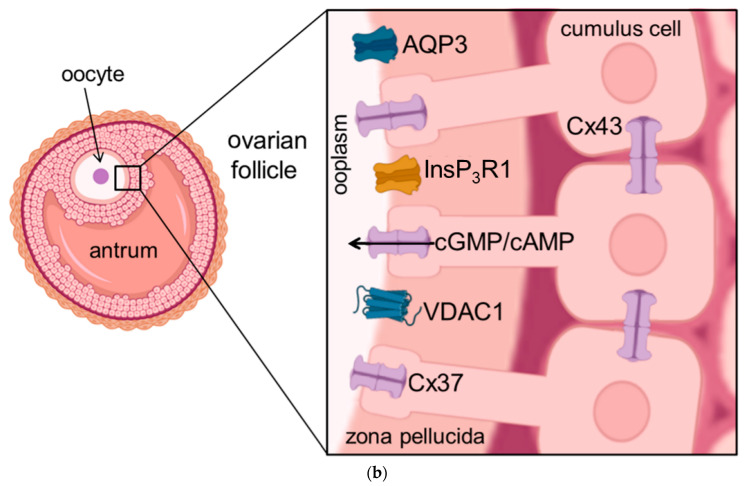
Schematic illustration of the cellular location of aquaporins and related proteins. (**a**) shows the localization and function of aquaporins and related proteins; (**b**) shows the localization of aquaporin and related proteins in the oocyte-cumulus-complex. Abbreviations: AQP—aquaporin; Cx—Connexin; CFTR—Cystic Fibrosis Transmembrane Conductance Regulator; Panx—Panexin; InsP3R1—Inositol trisphosphate receptor 1; VDAC1—Voltage dependent anion channel 1; ER—Endoplasmatic Reticulum; cGMP—cyclic Guanine Monophosphate; cAMP—cyclic Adenosine Monophosphate, ATP—Adenosine Triphosphate.

An important milestone in the study of water fluxes through biological membranes was the discovery of an aqueous pore serving as a specific water channel (Figure 1), today, known as aquaporin (AQP). In 1992, Peter Agre et al. described this structure for the first time in erythrocytes, and it was termed Aquaporin-1 (AQP1) and proved to be a paradigm shift in the knowledge of molecular and trans-membrane water transport [1]. Since water is the main and essential component in a wide variety of cells, AQPs are very important since they are able to increase the water permeability of cell membranes [1,2]. Water movements through cell membranes are important features for the osmoregulation and water homeostasis of a cell [3,4]. It is well-known that biological membranes with their hydrophobic character of the lipid bilayer have an intrinsic permeability for water due to their lipid composition [5]. Since the first discovery of AQP1 until today, a total of thirteen AQP isoforms have been identified in humans (AQP0–12) and are all classified as membrane channels that contribute to the permeation of water through membranes, due to osmotic gradients [6,7]. To date, the mRNA or protein expression of the thirteen human aquaporin isoforms have been described in numerous organs and tissues. The classification of human AQPs into three groups is based on the primary structure and permeation abilities of AQPs [8,9,10]. In general, the architecture of AQPs in cell membranes can be described as homo tetrameric and each monomer constitutes a pore, which is functionally independent [11]. The discovery of AQPs started with ground-breaking experiments in 1992, where a glycosylated component of a 35–60 kD protein of human erythrocytes was described on the electrophoretogram. Only few years later, a new integral membrane protein of human erythrocytes was described, which was composed out of a non-glycosylated component (28 kD) and a glycosylated component (35–60 kD). This functional unit of a membrane water transporter was named “CHIP28” (channel-forming integral protein). However, in 1993, CHIP28 was renamed AQP1 by Agre et al., who were Nobel Prize laureates in chemistry for the discovery of water channels [12,13] in 2003. Since that time, over the last three decades, AQPs have been described as being present in several organ systems, and in this paper, their importance for the female reproductive system is elaborated.

## 2. Aquaporins in the Female Mammalian Reproductive System

Previous studies have provided strong evidence, that at least eleven aquaporin isoforms, i.e., AQP 1, 2, 3, 4, 5, 6, 7, 8, 9, 11, and 12, have been identified in the female reproductive tract of different mammals, including the human, ovine, canine, and porcine species, and rodents (Table 1). The first aquaporin in the female reproductive system was confirmed by isolating the complementary DNA encoding for a water channel generated from a human uterus. In this first report, the cloned cDNA appeared with a high (99.8%) homology to the 28 kDa human erythrocyte CHIP28 which was earlier mentioned [13]. Further, Li et al., who investigated the cDNAs of CHIP28 and uterus AQP, showed that the primary structures deduced from the cDNAs show 99% identity and the only difference is an alanine to valine substitution at position 45 of the human CHIP28 [14,15]. Some years later, the localization of AQP1 in rat uterine tissue was confirmed by mRNA expression [16]. In general, aquaporins in the female reproductive system appear to be involved in water movement at an intraluminal, interstitial, and capillary level, and their expression seems to be regulated by steroid sex hormones e.g., progesterone [17,18,19,20]. Due to these results provided by numerous research groups, aquaporins appear to be important for the female reproductive physiology, which will be discussed in the following section in a more detailed fashion.

### 2.1. The Expression of Aquaporins in the Vagina

As shown in Table 1, the abundance of AQP1–6 and AQP10–12 has been so far reported in the vagina, and the main role of AQPs in this part of the female reproductive tract is considered to be vaginal lubrication [21,48]. In pre-menopausal women, AQP1 appeared to be mainly localized (after immune-labelling) in the small blood vessels of the vaginal wall, i.e., in the capillaries and venules [21]. The proteins AQP2, 5, and 6 were immuno-localized in the cytoplasm of the vaginal epithelium, whereas the AQP3 protein was mainly detected in the plasma membrane of the vaginal epithelium [21]. Further, AQPs have also been detected in rat vagina [43,48]. When compared to the human species, rat AQPs show similar characteristics: similar protein localization, AQP1 in the rat vagina is localized in small blood vessels of the vaginal wall, AQP2 was detected in the cytoplasm of the vaginal epithelium, and AQP3 was also immune-localized in the plasma membrane of the vaginal epithelium [43,48]. Another study on intermediate layer cells of the murine vaginal epithelium provided strong evidence that AQP3 was detected in their plasma membrane [49]. Additionally, AQP4 was immune-localized in the basolateral membrane of superficial layer cells in the murine vaginal epithelium [49]. In summary, aquaporins appear to be mainly relevant for the moisture environment of the vaginal mucosa.

### 2.2. Aquaporins and the Functioning of the Ovary 

Interestingly, AQP1 localization in the ovary is comparable to its localization in the vagina, i.e., in the microvascular and in the epithelial cells of small blood vessels, and its expression is rarely present in ovarian tumor cells [32,33]. The relative mRNA abundance for AQP1, 2, 3, and 4 was investigated in human ovarian follicles. More precisely, the expression of these four AQPs was present in theca and granulosa cells (GC) and their expression seemed to be dependent on the time to ovulation [34]. Therefore, it was assumed that the relative mRNA expression of AQP1–4 in the human ovary is controlled by ovarian hormones. Furthermore, a previous study provided evidence that AQP7–9 are also expressed in ovarian follicles of rats, where they most likely play a role during follicular development since AQPs seem to be responsible for the trans-cellular movement of H_2_O to form the antrum in antral follicles [57]. It was also shown that the mRNA expression of AQP5, 7, 8, 11, and 12 was detectable not only in neonatal murine ovaries, but also in murine GC of pups at the age of four weeks [58,59]. Further, the expression of mRNA and proteins has been reported for AQP5, 8, and 9, which appeared to be localized in the epithelium of rat oviducts, and, more specifically, the immune-localization for AQP5 and 8 was revealed in the cytoplasm, and AQP9 was localized in the plasma membrane [56]. AQP1, 5, and 9 have been demonstrated in the porcine female reproductive system, i.e., in the ovary, oviduct, and uterus [26,27,28,29]. Interestingly, AQP1 was detected in the endothelium of the ovarian capillaries, whereas AQP5 expression was analyzed in cells of primordial follicles, in GC of developing follicles, and epithelial cells of the oviduct [26]. In this part of the female reproductive tract aquaporins appear to be mainly involved in the supply of fluid, which is crucial for follicular development and growth according to the physiological function of the estrous cycle. 

### 2.3. Aquaporins and the Functioning of the Uterus

The relevance of AQPs for the physiological function of the mammalian uterus as the crucial female organ of the reproductive tract is linked again to the vasculature, as already shown in other organs of this tract. The uterus is the major organ involved in feto-maternal communication, and fluid homeostasis during implantation, pregnancy, and early embryonic development [3]. AQP1 is highly expressed in the endothelium of uterine blood vessels [25]. Interestingly, AQP1 gene expression is much more abundant in capillaries and arteries compared to the same size veins of endometrial vasculature in women [44]. Contrary to the localization of AQP1, the expression of AQP2 was present in the glandular endometrial cells generated from women with physiological fertility [45]. Further, AQP3 was also reported to be expressed in the endometrium of women [47], and AQP3 was also highly abundant in human cervical cancer [68], but this will be presented in a more detailed fashion in the section related to female disorders. It has been reported that the AQP9 protein was localized in the cytoplasm of human oviductal epithelial cells [31]. Other studies have shown the expression of AQP1, 5, and 9 in the porcine uterus [27] and porcine oviduct [28] at different estrous cycle stages, namely at days 2–4, at days 10–12, and at days 14–16. Further, in the late stage of estrous cycle (days 18–20), there was also an expression detectable of AQP1, 5, and 9 in the porcine uteri [27] and oviducts [28]. It has also been assumed that the expression dynamics of AQP1, 5, and 9 in pigs appear to be influenced by the stages of the estrous cycle and early pregnancy due to hormonal composition [27,28,29]. Taking into account the uterine fluid homeostasis during the time of embryonic implantation, a fluid reduction has to take place during this crucial time to ensure the close contact of the early embryo to the superficial cells of the endometrium [69]. With regards to this, AQP 5 and 9 mediate the absorption of glandular fluid [3]. After implantation, placentation is also a crucial biological process, and the relevance of AQPs during this process will be reviewed in the following section. To sum up, aquaporins appear to be mainly responsible for creating the proper fluid micro-environment in the uterus and they contribute to the lubrication of the endometrium, which is crucial for sperm movement and implantation. 

### 2.4. Aquaporins and the Functioning of the Placenta

Numerous AQPs have been reported to be present in fetal membranes and are crucial during placentation and early stages of pregnancy. The haemochorialis placenta of the human species has shown a high relative mRNA abundance of AQP1, 3, 9, and 11 in the chorionic villi, whereas the mRNA abundance for AQP4, 5, and 8 was lower in the earlier mentioned part of the placenta [35]. Both gene and protein expression for AQP1, 3, 8, 9, and 11 have revealed the presence of these aquaporins in the human amnion and chorion during the entire length of pregnancy [36,37]. The relative mRNA abundance of AQP1 was reported to be in the placental vasculature [38] and AQP3 gene-expression was detected in the trophectoderm [37]. Further, the localization of the AQP3 protein in the human placenta has been reported in epithelial cells of the chorion and amnion [70]. Evidence has been provided that the expression of AQP4 was decreased in cells of the syncytiotrophoblast, but endothelial and stromal cells of placental villi collected in the first and third trimester of pregnancy showed an increase in AQP4 expression [71], which suggests that the expression of AQP4 appears to be pregnancy stage-dependent. AQP8 and AQP9 have been localized to the epithelium of the human amnion [60] and AQP9 was further shown to be present in trophoblast cells, in cytotrophoblast cells, and syncytiotrophoblast cells of the chorion [65,66]. During the phase of implantation and early placentation, the expression of AQP1, 5, and 9 have also been detected in the porcine species [27,28,29]. 

## 3. Aquaporins and Reproductive Aging 

**Figure 2:**
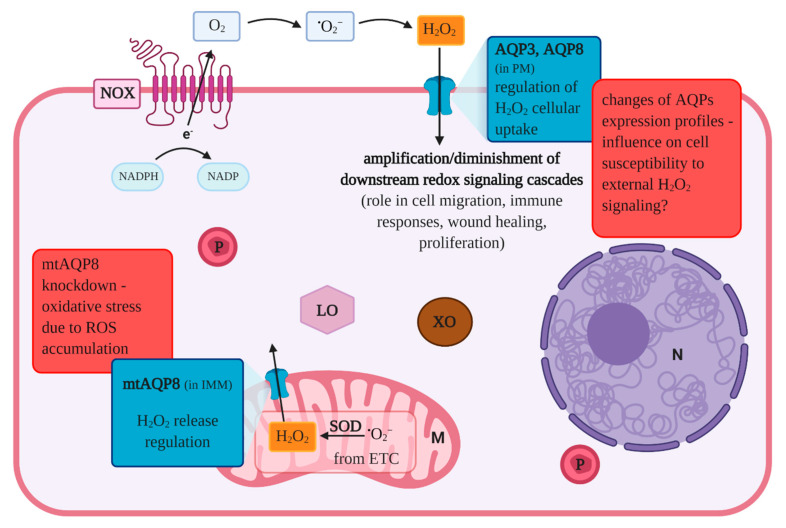
The role of aquaporins in the transport of reactive oxygen species and oxidative metabolism. The main physiological sources of reactive oxygen species include: mitochondria, nicotinamide adenine dinucleotide phosphate oxidase, xanthine oxidase and lipoxygenases. AQP3 and AQP8 belong to the family of aquaporins and can be classified as aquaglyceroporin and orthodox aquaporin, respectively. A growing body of research has demonstrated the involvement of these two membrane channels in mediating hydrogen peroxide cellular uptake, as described in the text. AQP-regulated H_2_O_2_ accumulation can amplify or diminish signal transduction pathways in which this molecule serves as a second messenger. As shown in other cell types, AQP3 and AQP8 expression changes influence complex biological processes, such as immune responses, proliferation, wound healing or cell migration. AQP8 was also detected in the inner mitochondrial membrane and its deregulation may be implicated in ROS accumulation which leads to mitochondrial depolarization and a reduction in ATP production. Therefore, aquaporins may play a role in oocyte oxidative metabolism changes observed with aging. Abbreviations: AQP—aquaporin; e—electron; ETC—electron transport chain; IMM—inner mitochondrial membrane; NADPH—dihydronicotinamide adenine dinucleotide phosphate; NADP—nicotinamide adenine dinucleotide phosphate; N—nucleus; M—mitochondrion; P—peroxisome; LO—lipoxygenase; XO—xanthine oxidase NOX—NADPH oxidase; PM—plasma membrane ROS—reactive oxygen species; SOD—superoxide dismutase, (according to [88,98,99,100,101,102,103,104,105,106] and this review).

Female reproductive aging in numerous mammalian species is linked to a progressive decline of the ovarian function, where a decrease in the quantity and quality of oocytes with advancing age has been reported. The female reproductive system is one of the first organ system to show symptoms of aging in comparison with other organs. However, the molecular mechanisms underlying the reproductive aging processes of oocytes need further elucidation [72,73,74,75]. In humans, this decline of women’s fertility also has implications for society, since the number of first pregnancies, at an advanced age, has increased significantly in most industrialized countries [76]. This delay is due to prolonged education, career ambitions, and awaiting financial security and stable relationships [77,78,79]. Although numerous assisted reproductive technologies are well-established nowadays, they are not always successful and require substantial financial investment, too [80]. It is well-known that oocytes, generated from women of advanced age (≥35 years), show an increased risk after fertilization of miscarriage and/or aneuploid offspring [80]. Oocytes produce energy predominantly through oxidative phosphorylation since glycolysis in the oocyte is possible only with limitations due to the low content of phosphofructokinase [81]. Previous research provided evidence, that the ATP content of an oocyte is related to its developmental competence [82,83,84], and mitochondrial dysfunction was shown to be related to oocyte maturation arrest, chromosomal misalignment, and reduced embryonic development [85,86,87]. However, during the ATP production, reactive oxygen species (ROS) are generated as a by-product, and there is a chronic exposure to ROS while oocytes are arrested in prophase I prior to ovulation. Due to the fact that chronic exposure to ROS can damage not only DNA but also lipids and proteins, the ‘Oxidative Stress Theory of Aging’ was introduced, which suggests that a progressive accumulation of oxidative damage results in a reduction of oocyte quality with advancing age. Taking into consideration aquaporins and aging, it has been reported that AQP8 and some other members of the mammalian AQP family facilitate H_2_O_2_ passage across plasma membranes, and it has been shown that AQP3 is required for (NOX)-derived H_2_O_2_ signaling [88]. More recent studies have provided evidence that AQP8 transports NOX-generated H_2_O_2,_ which is involved in intra-cellular signal transduction pathways [89,90,91]. These latter-mentioned functions are visualized in Figure 2, which shows the role of aquaporins in the transport of reactive oxygen species and oxidative metabolism. With regard to the importance of mitochondria for the reproductive aging, it is worth mentioning that AQP8 is also expressed in the inner mitochondrial membranes [92]. Further, it was suggested in a previous study that mtAQP8-mediated H_2_O_2_ transport might play a role in human spermatozoa [93]. Interestingly, the knockdown of mtAQP8 expression in HepG2 cells resulted not only in a reduction of H_2_O_2_ release generated in mitochondria but also in mitochondrial depolarization due to ROS accumulation and reduced ATP levels [94,95]. Therefore, it is not surprising that the quality of oocyte mitochondria is determining the quality of the oocyte, too [96]. In murine oocytes, the expression of AQP3 was detected where it appears to be responsible for the water permeability increase with low activation energy, and AQP3 has also been reported to be crucial for the permeability of low molecular weight nonelectrolytes. In another study, the APQ3 protein was localized via immunofluorescence in the plasma membrane of oocytes, which was intended to be shown in Figure 1 in combination with the importance of aquaporin-related proteins [53,97].

## 4. Aquaporins in Female Reproductive Tract Disorders

Comparable to the importance of aquaporins for female reproductive physiology, they also contribute to several female reproductive disorders, e.g., polycystic ovary syndrome, ovarian and cervical cancer and endometrial diseases. The extent to which aquaporins are involved in all of these mentioned pathologies will be described in detail in the following sections.

### 4.1. Polycystic Ovary Syndrome (PCOS) 

PCOS is a common and complex endocrine disorder in women, which could lead to infertility. It is estimated that approximately 10% of the total female population has PCOS. Other studies indicate that 70% of infertility in women and about 40% of miscarriages are due to the presence of PCOS [107]. PCOS is described as a systemic disease with multifaceted symptoms, for instance, disorders of the menstrual cycle, increased levels of androgens and anovulation, irregular menstrual cycles, hirsutism, numerous metabolic abnormalities in the form of obesity, dyslipidemia, and insulin resistance. Studies suggest that 3 out of 12 AQP isoforms show altered expression in PCOS (AQP7, 8, 9) [108]. Despite knowledge of this disease, its pathogenesis has not been fully characterized. Wawrzkiewicz-Jałowiecka et al. suggest that PCOS is a systemic disease caused by a set of various mutations [109]. These mutations cause, among others, overexpression of AQPs [109]. Research indicates that there may be cause-effect relationships between the expression of AQP 7–9 in adipocytes and GCs and the symptoms of PCOS [110]. The high level of androgens in PCOS patients leads to reduced AQP9 expression, as well as impaired function in GCs, thus, hindering follicle development [105]. The use of modulators to lower the expression of AQPs (especially AQP7 and AQP9) may improve glycerol metabolism and indirectly improve ovulation by reducing the level of androgens. Studies conducted at the mRNA level indicate a significantly higher expression of AQP8 and a significantly lower expression of AQP9 in the ovarian tissues of patients with PCOS compared with the control sample [111]. It appears, therefore, that the above scheme may become a biological indicator of the disease. 

Depending on the conducted studies, it is suggested that changes in AQP9 expression may affect the normal development of ovarian follicles, which may be related to the clinical symptoms of PCOS [105,112]. Lu et al. did not find any significant differences in the expression of AQP9 in luteinized GCs from PCOS women compared to the control sample (women with normal follicle development, referred to the in vitro fertilization (IVF) procedure due to obstruction of the fallopian tube) [113]. Moreover, they found no correlation between the level of AQP9 expression in the GCs layer and the level of E_2_ (Estradiol), P_4_ (Progesterone) in the follicular fluid [113]. Identifying the function of AQP in the ovary of women, especially in the GCs, also provides better insight into the pathophysiology of PCOS. In further research, it seems important to pay attention to the problem of insulin resistance and obesity in PCOS. It should also be investigated whether altered pH in insulin resistance affects the expression and transport properties of AQPs. Moreover, it is worth examining whether the increased level of androgens correlates with the expression of AQPs. These results will help to identify the role of AQPs in identifying the causes and treating symptoms of PCOS.

### 4.2. Ovarian Cancer 

AQPs are primarily responsible for cell proliferation, migration, and adhesion, as well as for angiogenesis of healthy tissues. Nevertheless, AQPs can also be expressed in cancer tissues. The presence of one type of AQPs in several types of cancer makes it impossible to select specific AQPs as molecular markers of particular types of cancer. The results of studies on the expression of AQP1 in vascular endothelial cells indicate a key role of AQP1 in tumor angiogenesis by accelerating the migration of cancer cells. *AQP1* knockout mice showed low angiogenesis in the cancer tissue resulting in subcutaneously induced melanoma tumor necrosis in these mice [114]. However, the overexpression of *AQP1* in cancer tissue resulted in strong migration, invasion and metastasis of cancer cells to other organs [115,116]. The above property presents a new face of AQP1, thanks to which it may become a potential target for the development of anti-cancer drugs. AQP1 may also contribute to the high permeability of blood vessels and be responsible for the formation of exudates and edema fluid. Mobasheri et al. observed a slight increase in ovarian cancer tumor tissue expression and a significant increase in advanced breast cancer [117]. 

In malignant forms of ovarian cancer, a much higher expression of *AQP1*, *AQP5*, *AQP9* was observed [118] compared to benign forms of ovarian cancer or normal ovaries. Ovarian cancer is a very complex disease with a high death rate among women [119]. Late detection gives little chance of a complete recovery. Advanced ovarian cancer is associated with ascites. One of the causes of ascites is an imbalance in water transport, which is the result of changes in the expression of AQPs [120,121]. AQP1 has been shown to be present in the microvascular endothelium of ovarian tissue but rarely in ovarian tumor cells [120]. Moreover, AQP1 was localized mainly in vessels and microvessels, not in cancer cells. There is also a positive correlation between AQP1 expression and the occurrence of ascites and the progression of an ovarian tumor [121]. Overexpression of AQP9 was characteristic not only of normal ovarian superficial epithelium but also of malignant ovarian tumors. As with AQP9, high AQP5 expression was characteristic of malignant ovarian tumors associated with lymph node metastases. AQP5 protein has been located in the basolateral membrane of the epithelial layer in benign tumors and plasma membranes of borderline and ovarian tumors. A change in AQP5 expression was also noted in the ovarian cancer cell line CAOV3 and SKOV3. In the first case, the use of cisplatin reduced the expression of AQP5 and the rate of tumor cell proliferation [122]. When treated with epigallocatechin gallate, the SKOV3 cell line showed reduced AQP5 expression while inhibiting the proliferation of tumor cells [123]. Expression of AQP5 was evident in GCs and theca cells (TCs) in normal ovaries while immunohistochemistry revealed the presence of AQP5 in surface epithelium, fibroblast cells of the stroma and cells lining tumor and acini. Western Blot analysis showed higher AQP5 concentrations in cancerous ovaries compared to healthy ovaries. 

Yang et al. characterized the presence and localization of individual AQP subtypes in ovarian epithelial carcinomas [124]. Each of the AQP subtypes expressed a different pattern of expression and a different localization. As in the above-mentioned studies, AQP1 was expressed mainly in the microvascular endothelium, and AQP2–9 in cancer cells. AQP1, 5, and 9 expression was significantly higher in malignant tumors than in benign tumors [124]. The immunohistochemical studies showed that AQP6 expression was significantly lower in malignant tumors than in benign or normal tissue. Moreover, high AQP1 expression was correlated with the occurrence of ascites in patients with ovarian malignancy [120]. Research indicates that AQP1, 3, 5, and 9 expression may become useful biological markers in ovarian cancer prognosis, but their correlation with a prognosis depends on the type of cancer present [125].

### 4.3. Cervical Cancer 

Cervical cancer is another one of the most common causes of death from cancer in women. In recent years, early diagnosis has increased significantly, especially cervical smear tests in developing countries [126]. The overexpression of AQP is characteristic of many types of human cancers, but their role in cervical cancer has not yet been precisely defined [126]. Cervical cancer has also been reported to be associated with an altered pattern of AQPs expression. As in the case of ovarian cancer, most studies focus primarily on the expression of AQP1, 3, 5. Molecular studies can give a large prognostic value to these proteins [127,128]. Chen et al. observed the overexpression of only two AQP subtypes (1 and 3 in cervical cancer) and also analyzed the correlation between AQP1 and 3 expression and prognosis in cervical cancer [127]. The expression of AQP 1 and 3 in cervical carcinoma, cervical intraepithelial neoplasia, and normal cells was compared by RT-PCR, immunohistochemistry and immunofluorescence. AQPs showed different expression in both the mRNA and protein level in different cell types. AQP1 was localized in the tumor vessels, while AQP3 showed increased expression in cervical cancer, compared to intraepithelial neoplasia, and normal cells [127]. It was also noticed that the expression of AQP1 and AQP3 was increased in the advanced stage of cancer, the larger tumor, in patients with metastases, which correlates with the patient’s prognosis. The results of Chen et al. clearly indicate that AQP1 and AQP3 are associated with the progression, development of vascularization, and metastasis of cervical cancer [127]. Other studies have demonstrated that tumor angiogenesis in AQP1 knockout mice after tumor xenograft was clearly inhibited [114]. Zhang et al. also observed increased expression of AQP5 mRNA and protein during the proliferation of cancer cells in cervical cancer. Overexpression correlated with lymph node involvement. AQP5 was also found to correlate positively with the Ki-67 proliferation index. Analysis of the survival rate of patients with AQP5 and Ki-67 overexpression was associated with a much worse prognosis [129]. Another result concerning the expression of AQP1 in cervical cancer was presented by Wei et al. [126] who demonstrated decreased expression of AQP1 mRNA and protein in cervical cancer. AQP1 was positively expressed in normal healthy tissues. The decreased expression correlated AQP1 with progressive symptoms characteristic of cervical cancer [126]. Shen et al. examined the expression of AQP1, 3, 4, 5, and 8 in cervical intra-epithelial neoplasia (CIN), squamous cervical cancer (SCC) and normal cervical tissues [130]. AQP3, 4, 5, and 8 expression was higher in SCC than in normal tissues. Expression of AQP3 and 8 was correspondingly higher in SCC than in CIN. AQP4 expression was higher in CIN than in normal cervical tissues. There were significant changes in AQP1 and 3 expression at different tumor stages [130]. Despite many studies, the role of AQPs in human cervical cancer is still not fully defined. The only observed AQPs with increased expression are AQPs 1 and 3. However, further studies are needed to determine the role of AQPs in the diagnosis and prognosis of cervical cancer in women. These observations suggest that AQP5 plays one of the key roles in the development of cervical cancer. At the same time, according to the authors of the above studies, it may become a new therapeutic target, and at the same time, a prognostic marker for this disease. 

### 4.4. Endometrial Diseases

Endometrial cancer is related to the development of a tumor in the lining of the uterus (the endometrium). Zou et al. observed that AQP2 expression levels were low in the early stages of the disease [131], while Jiang et al. noted that AQP5 expression increased in the later stages of the tumor [132]. In adenocarcinoma and endometrial hyperplasia, AQP1 was localized in the microvascular epithelium and small vessels of the tumor. Depending on the type of disease, the ratio of AQP1 expression to intra-tumor microvessels was the highest in adenocarcinoma. The amount of expression was also correlated with the severity of the disease, including ectopic metastases [103]. Blocking AQP5 expression reduced cell migration of this tumor [133]. As mentioned previously, AQP is responsible for the migration of cancer cells, and thus changing the shape of the cells and their volume can promote tumor metastasis [134]. It is known that AQP5 expression depends on the E_2_ (estradiol) level. A study by Jiang et al. provided evidence that elevated AQP5 expression is present in endometrial cancer as well as endometriosis [135]. AQP5 is also present in physiological tissues, as well as in endometriosis. It was also determined that the amount of AQP5 expression in the endometrium depends on the phase of the menstrual cycle [136]. The above-mentioned studies show that the expression of individual AQPs correlates with the patient’s prognosis and with the cancer stage. Most of the described AQPs in ovarian, cervical or endometrial tumors show increased expression. At the same time, the migration and proliferation of neoplastic cells may be dependent on the expression of AQP.

## 5. Aquaporins and Related Proteins

For fluid homeostasis and proper functioning of eukaryotic cells, aquaporin-related proteins are of specific relevance, particularly for communication between cells, movements of specific ions, adenosine triphosphate and second messengers (Figure 1). In addition, previous reports indicated that the normal function of AQP is linked to the expression of other proteins. The following section elucidates how important aquaporin-related proteins are for the functioning of the female reproductive tract. There are six main types of ion channels: (1) Sodium channels; (2) Calcium channels; (3) Potassium channels; (4) Chloride channels; (5) Porins and (6) Gap junction proteins [137,138,139] (Table 2). Calcium channels have a selective permeability to calcium ions. Within this family, 3 out of 5 groups can be distinguished, closely related to the proper functioning of the reproductive system and proper fertilization [140,141]. Further, there are cation channels, which are associated with spermatozoa (also known as CatSper 1, 2, 3, 4). When sperm enter the alkaline environment of the female reproductive system, the concentration of ions in the sperm flagella changes. Therefore, it can be assumed that these channels are responsible for proper fertilization [142]. TPCN1 and 2 proteins are closely related to CatSper of the sperm tail [143,144,145]. 

Another group of proteins showing a relationship with AQPs is inositol triphosphate (InsP3R). This membrane glycoprotein complex acts as a CA^2+^ channel and its activation is mediated by inositol triphosphate (InsP3). It is responsible for a number of physiological processes, including: Proper fertilization, cell proliferation, and cell division [146,147,148]. The preservation of the proper functioning of the reproductive system is also provide by the chloride channels, ATP-gated CFTR. It is an anion channel regulated by cAMP-dependent phosphorylation found in many tissues, including the reproductive system [149,150,151]. CFTR gene mutation can cause cystic fibrosis, chronic lung disease, and infertility [152]. Channels formed by CFTR proteins are responsible, under physiological conditions, for the proper passage and secretion of fluids within the reproductive system (concentrate sperm, fluid secretion in the seminiferous epithelium, luminal fluid in oviduct) [152]. These proteins are located in various parts of the reproductive system of both animals and humans. CFTR expression is dependent on ovarian hormones and at the same time influences the volume of fluids in the female reproductive system [153]. Like AQPs, voltage-dependent anion channels (VDAC) belong to the porin family. The role of AQPs in both the female and male reproductive systems has been partially explained and described in the literature by many authors [102,111,115]. The second family of porin genes includes genes responsible for the expression of three different proteins: VDAC1, 2, 3 [154,155,156]. VDACs form hydrophilic pores that allow metabolites to pass through the outer mitochondrial membrane, and are involved in metabolite transport, signal transduction, fatty acid ions and Ca^2+^ transfer [157,158,159]. It has also been suggested that these proteins are found in extra-mitochondrial membranes [137]. VDAC expression, otherwise known as mitochondrial porins, is important in sperm function [160]. VDAC is located in Sertoli cells while VDAC2 has been localized in the acrosomal region and principal piece, and in late spermatocytes, and spermatids. VDAC3 has been localized in the acrosomal region and mid-piece, and all cell types of the testis (mainly Leydig cells) [161,162,163]. Blocking VDAC expression with DIDS significantly reduced the quality of sperm and significantly limited their mobility, viability and fertilization [160]. The first reports of VDAC expression in porcine oocytes appeared in 2009. Cassara et al. identified the presence of VDAC1 and 2 in porcine oocytes (GV-germinal vesicle, MII—meiosis II). The VDAC1 protein was located across the entire surface of the oocyte [137]. However, little is known about the expression and localization of VDAC in gametes and the mammalian reproductive system. Further research on these proteins may serve as non-hormonal contraceptives for men and women. The role of VDAC in the maturation of mammalian oocytes has not yet been fully established.

Another group of transmembrane proteins responsible for intercellular communication are gap junction proteins also called connexins (Cxs). These proteins form gap junction connections (GJCs) [164,165,166]. GJCs were discovered 40 years ago [167,168], and in recent years, it has been found that they interact with other membrane channels to maintain homeostasis in specific tissues. The interactions between connexins and other transport channels may be direct or indirect, depending on the type of proteins involved in transport. These are channels enabling the exchange of ions as well as small metabolites (1–2 kDa). Recent studies indicate the significant role of Cxs in the reproductive system, as well as in the maturation and development of development skills by the oocyte [166,169,170,171]. Proteins that build cell-cell connections are becoming increasingly important in the proper functioning of individual systems, including the reproductive system. It seems that their individual types have been well-known and described in the literature. Quite a new and still unknown area of knowledge is the study of dependencies and interactions between particular types of connections. The first evidence of an interaction between the GJCs and other transport channels appeared in cystic fibrosis research. Mutations in the *CFTR* gene cause water and dissolved substances to pass through cells inadequately, which are symptoms of the disease. It has also been suggested that the *CFTR* gene, as already mentioned, is expressed in many tissues, not only epithelial cells [167,168]. Johnson et al. suggested that GJCs play a significant role in enhancing the functional effects of cells in correcting cystic fibrosis by inserting the wild-type *CFTR* gene [172,173]. Chanson et al. suggested that the malfunction of the GJCs is related to the tissue malfunction in cystic fibrosis. This indicates some kind of relationship between GJC and the presence of mutations in *CFTR* [174]. Many studies suggest that CFTR plays an important role in the Cxs gating mechanism, which affects the voltage sensitivity of a given channel. Kotsias et al. also suggest that cytoplasmic proteins may play a significant role in the CFTR-Cx interaction, e.g., in *Xenopus* oocytes [175]. It has been suggested that the mechanisms of these interactions are not fully understood and elucidated in many tissues, especially not in the reproductive system [174,175]. The research on functional relationships between AQPs and Cxs was carried out using lens because it has no blood supply. Delivering and/or exchange of electrolytes and metabolites takes place via GJCs. The structural proteins of junction of chicken lens epithelial cells are Cxs, especially Cx43, Cx45.6, Cx56 [106,176]. Studies by Yu and Jiang using confocal microscopy clearly indicated the co-localization of the above proteins in the lens of a chicken embryo [177,178], as well as their cooperation in intercellular communication. Interactions and complications in Cxs and AQPs have also been observed in mouse brain astrocytes [179,180] and during postnatal neurogenesis [181]. In the perivascular membranes of astrocytes, AQP4 is responsible for water transport, while Cx43 is a protein of the gap junctions. In studies of murine astrocytes, Nicchia et al. noticed that AQP4 gene silencing is closely associated with a decrease in Cx43 expression [180]. Although it was concluded that Cx43 mediates the regulation of the flow of ions and water, the molecular mechanism of the interaction is unknown [180,181]. Other studies on postnatal neurogenesis also indicate the expression of Cx43 and AQP4 in the ventricular zone (VZ) cells [182]. 

Interestingly, it is believed that there three types of “large pore” channels (AQPs, Cxs, Panxs; Pannexins). The function of these channels is most likely regulated in three ways; regulation of solute gradient; cytoskeleton signalling related to changes in cell volume, and; nucleotide signalling. The flow of individual ions through Cx43 and Panx1 most likely depend on the ability of AQP4 to regulate the solute concentration gradients. Changes in cell volume during proliferation are related to the movement of water and the activity of AQP4 inside the cell, which is related to changes taking place in the cytoskeleton of nerve cells [181]. Panx1, on the other hand, most likely mediates the release of ATP from cells [181]. It has also been proven that Cx30 is involved in maintaining water and ion homeostasis in the nervous tissue [183]. Deletion of both Cxs (Cx43 and Cx30) leads to a reduction in expression of total AQP4 protein [183]. Although other studies have indicated an important role of Cx43 and AQP4 in the formation of cerebral edema, the authors of those studies indicate that the mechanism of interaction and mutual correlations between the two types of channels is not fully understood [184]. Correlations between different types of intercellular connections were also noted during myocardial edema after cardiopulmonary bypass. These symptoms are related to the high expression of AQP1, which affects the amount of Cx43 expression during the onset of myocardial edema [185,186]. Communication between the oocyte and cumulus cells (CCs) is possible through GJC (Figure 1). Although the role of Cxs in the reproductive system has been thoroughly described by many authors [187,188,189,190], increasing attention has been paid in recent years to the role of Panxs in the reproductive system. These proteins forming single membrane channels allow the cytoplasm to contact the extracellular environment [191,192,193]. Dye et al. found differential expression of Panx1 in COCs of bovine isolated vesicles of various sizes and suggested the involvement of Panx1 in the process of oocyte maturation [194]. It has been shown that the *PANX1* gene mutation causes the degeneration of oocytes since they release much more ATP into the extracellular space [195]. Other studies suggest a close relationship between the occurrence of AQP and Cx. in porcine luminal epithelium cells. Wojtanowicz et al. investigated this relationship in a short term (7 days) in vitro culture. The expression of 10 AQPs (AQP2, AQP3, AQP4, AQP5, AQP6, AQP7, AQP8, AQ9, AQP10, AQP11) and 4 Cxs (Cx36, Cx37, Cx40, Cx43) and linking their expression to a real-time proliferation assay were examined. The tests were performed on endometrial cells taken from the porcine uterus. The expression of individual AQPs and Cxs was examined at the mRNA and protein levels [196]. Although, all analyzed Cxs and AQPs were shown to be present, they showed different expression patterns at different culture time intervals. Expression studies at the transcript level indicate an increase in the expression of Cx37, Cx40 and Cx43 as well as AQPs in relation to the starting point of the culture (24 h—reference value). The ultrastructure of the endometrial cells showed changes during the menstrual cycle. A large role in these processes is assigned to GJC and water channels. Due to these connections, cells can quickly react to hormonal and nervous stimuli, as well as changes in water volume [196]. The results of the research by Wojtanowicz et al. indicate that the determination of the expression of AQPs and Cxs in endometrial epithelial cells may be an important indicator in the development of the endometrium, and may also be related to its function and changes occurring during the menstrual cycle in humans.

## 6. Conclusions

Aquaporins have been intensively investigated in the female reproductive system since their discovery in the uterus. Further studies have identified the expression of at least eleven AQP isoforms in the female mammalian reproductive system. These expressions have been detected by molecular biological and pharmacological methods in different species. AQPs are one of the best-characterized membrane protein families, which enables a particular understanding of their basic mechanism activity, due to substrate specificity and the regulation of the characterized membrane proteins above. Moreover, other proteins may participate in maintaining proper cellular homeostasis in biological cooperation with AQPs. This paper analyses the research results which have been provided in the past to provide an interesting update for other research groups who are working on aquaporins in relation to the female reproductive tract. This review serves as a resource for future research projects seeking for further elucidate further the interaction of AQPs, related to the physiology, pathology, and aging of the female reproductive system in mammals.

## Figures and Tables

**Table 1:** Overview of the expression of aquaporins (AQP) at mRNA and/or protein levels in the female reproductive tract of different mammalian species. Numbers in brackets indicate the references.

AQP	Vagina	Cervix/Cervical Carcinoma	Uterus	Oviduct	Ovary	Follicle/Oocyte	Embryo/Amnion/Chorion
**AQP1**	Human [21] Rodent [22,23]	Human [24]	Human [25] Rodent [19] Porcine [26,27,28,29] Canine [30]	Human [31] Rodent [22] Porcine [26,27,28,29]	Human [32,33,34] Porcine [26,27,28,29]	Porcine [26,27,28,29]	Human [35,36,37,38,39] Rodent [40,41] Ovine [42]
**AQP2**	Human [21] Rodent [43]		Human [25,44,45,46] Canine [30]	Human [47]		Human [34]	Human [39]
**AQP3**	Human [21]	Human [24]	Human [47]			Human [34]	Human [35,36,37,38,39]
	Rodent [43,48,49]	Rodent [50,51]				Rodent [52]	Rodent [40,41,53,54]
							Ovine [42]
**AQP4**	Rodent [49]	Rodent [50]	Rodent [19]			Human [34]	Human [35,36,37,38,39]
**AQP5**	Human [21]		Rodent [19,55]	Rodent [56]	Porcine [26,27,28,29]		Human [35,39] Rodent [41]
	Rodent [48]		Porcine [26,27,28,29]	Porcine [26,27,28,29]			
			Canine [30]				
**AQP6**	Human [21]						Rodent [41]
	Rodent [48]						
**AQP7**			Rodent [55]			Rodent [57,58,59]	Human [39] Rodent [41,54]
**AQP8**		Humans [24]	Rodents [19,55]	Rodent [56]		Rodent [57,58,59]	Human [35,36,60,61,62]
		Rodent [50]					Rodent [40,41,52,63,64]
							Ovine [42]
**AQP9**			Rodent [19,55]	Human [31]	Porcine [26,27,28,29]	Rodent [57]	Human [35,36,65,66]
				Porcine [26,27,28,29]			Ovine [67]
**AQP10**	Rodent [48]						
**AQP11**	Rodent [48]						Human [35,39]
							Rodent [54]
**AQP12**	Rodent [48]				Rodent [58]		Human [39]

**Table 2:** The main types of ion channels and related proteins in the reproductive system. Numbers in brackets indicate the references.

Type of Ion Channel	Type of Protein	Location	Function
**Porins**	VDAC1, 2, 3	Sertoli cells [162]; GV (germinal vesicle) and MII (meiosis II) stage porcine oocytes [137] outer dense fibers of the bovine sperm flagellum; head of bovine sperm, late spermatocytes, spermatids and spermatozoa of the bovine testis [161]; GV (germinal vesicle) and MII (meiosis II) stage porcine oocytes [137]; mouse granulosa cells [197] outer dense fibers of the bovine sperm flagellum in porcine [161];	participation in follicular development and autophagy suppression to folliculogenesis in mammals [197]; deficient males are infertile because of structural abnormalities in the sperm tail, leading to sperm immotility [198]
**Cation channels sperm associated**	CATSPER1, 2, 3, 4	Plasma membrane of the sperm tail [144] testis [199]	key role in the motility, hyperactivation and fertilization function of sperm [141,200]
**Inositol trisphosphate receptor**	InsP3R1, 2,3	human GCS [146] mouse oocyte [201]	proper fertilization [148]
	CFTR	rat epididymal epithelial cells [174]; porcine vas deferens epithelial cells [202]; vagina, cervix, uterus and fallopian tubes, in rodents and humans [153,203,204]; mouse endometrial cells [205]	CFTR plays a key role in regulating Cl^−^ secretion, and thus fluid volume in male and female reproductive tract [152,202]; sperm capacitation [206]
**Gap junction protein**	Cxs	mouse, human, rat, pig, dog seminiferous tubules [190]; mouse, human, swine, bovine, canine ovary [207,208,209,210]; oocyte and granulosa cells (GCs) [171,211]; human, mouse and baboon endometrium [170,212]	function as nurturing the germ cell lineage; developmental competence by oocyte, communication with cumulus oophorus cells; connection between GCs population, mural—mural GCs communication; folliculogenesis [213,214]
